# Molecular Responses to Small Regulating Molecules against Huanglongbing Disease

**DOI:** 10.1371/journal.pone.0159610

**Published:** 2016-07-26

**Authors:** Federico Martinelli, David Dolan, Veronica Fileccia, Russell L. Reagan, My Phu, Timothy M. Spann, Thomas G. McCollum, Abhaya M. Dandekar

**Affiliations:** 1 Dipartimento di Scienze Agrarie e Forestali, Palermo, Italy; 2 Euro-Mediterranean Institute of Science and Technology (IEMEST), Palermo, Italy; 3 Department of Plant Sciences, University of California Davis, Davis, California, United States of America; 4 California Avocado Commission, Irvine, California, United States of America; 5 United States Department of Agriculture-The Agricultural Research Service, U.S. Horticultural Research Laboratory, Fort Pierce, Florida, United States of America; Fujian Agriculture and Forestry University, CHINA

## Abstract

Huanglongbing (HLB; citrus greening) is the most devastating disease of citrus worldwide. No cure is yet available for this disease and infected trees generally decline after several months. Disease management depends on early detection of symptoms and chemical control of insect vectors. In this work, different combinations of organic compounds were tested for the ability to modulate citrus molecular responses to HLB disease beneficially. Three small-molecule regulating compounds were tested: 1) L-arginine, 2) 6-benzyl-adenine combined with gibberellins, and 3) sucrose combined with atrazine. Each treatment contained K-phite mineral solution and was tested at two different concentrations. Two trials were conducted: one in the greenhouse and the other in the orchard. In the greenhouse study, responses of 42 key genes involved in sugar and starch metabolism, hormone-related pathways, biotic stress responses, and secondary metabolism in treated and untreated mature leaves were analyzed. *TGA5* was significantly induced by arginine. Benzyladenine and gibberellins enhanced two important genes involved in biotic stress responses: *WRKY54* and *WRKY59*. Sucrose combined with atrazine mainly upregulated key genes involved in carbohydrate metabolism such as sucrose-phosphate synthase, sucrose synthase, starch synthase, and α-amylase. Atrazine also affected expression of some key genes involved in systemic acquired resistance such as *EDS1*, *TGA6*, *WRKY33*, and *MYC2*. Several treatments upregulated *HSP82*, which might help protect protein folding and integrity. A subset of key genes was chosen as biomarkers for molecular responses to treatments under field conditions. *GPT2* was downregulated by all small-molecule treatments. Arginine-induced genes involved in systemic acquired resistance included PR1, *WRKY70*, and *EDS1*. These molecular data encourage long-term application of treatments that combine these regulating molecules in field trials.

## Introduction

Plant pests and diseases threaten agricultural systems. Huanglongbing (HLB) disease endangers the citrus industry and citrus cultivation worldwide [1]. Neither genetic resistance nor short- or long-term therapeutic strategies to mitigate HLB has been found. Huanglongbing disease is associated with three *Candidatus liberibacter* (C. Las) species: *asiaticus*, *americanus*, and *africanus*. C. Las is a member of the alpha subdivision of the proteobacteria based on ribosomal region sequence data [2]. Symptoms have been extensively described and all citrus species are susceptible to HLB to varying degrees [3,4].

Microarray analysis identified key genes and pathways affected by HLB at the transcriptomic level in mature, symptomatic leaves [5,6]. RNA-seq was applied to describe molecular responses in fruit with different degrees of HLB symptoms [7]. This allowed a comprehensive analysis of gene regulatory networks on source and sink tissues at different developmental stages [8,9]. Effects of C. Las infection on key genes involved in sugar and starch metabolism, disrupting source-sink relationships, was a key cause of the metabolic dysfunction. Fruits of infected plants remained immature and photosynthesizing while mature leaves became yellow and accumulated starch [9]. Protein expression has been linked to the nutritional condition of grapefruit plants before and after symptom appearance. C. Las-upregulated proteins were involved in redox reactions, cell wall modification, and biotic stress responses [10]. Isobaric tags for relative and absolute quantitation (iTRAQ) identified which pathways are affected post-transcriptionally by pathogen infections [11]. A predictive proteome analysis of C. Las has been conducted [12]. Because no toxins or other pathogenic substances were clearly identified in the genome [13], the pathogenetic mechanisms of HLB disease are still unclear, nor is it clear whether the HLB-associated changes in sugar and starch metabolism are a cause or an effect of the disease [9].

Current management procedures consist mainly of visual scouting for symptoms, PCR-based detection of the pathogen, and insecticides for vector control [14]. Although application of insecticides can reduce disease spread, the disease can spread with only a few infected psyllids in the orchard. Early disease detection and psyllid control are critical practices in areas where neither disease nor vector has yet been discovered [1]. At present, no chemical compounds have been tested to beneficially modulate Citrus host responses and eventually extend the life and production of HLB-infected trees, reducing high economic costs due to lost production. Research has focused only on testing compounds targeting the pathogen. A combination of two antibiotics (penicillin and streptomycin) applied by either root soaking or foliar spray decreased C. Las titer in infected plants [15]. Antimicrobial compounds have been delivered through graft-based chemotherapy [16]. Nanoemulsion formulations were evaluated for their ability to increase permeability of antimicrobial molecules with success dependent on the citrus cultivar and degree of HLB symptoms [17]. Small-molecule inhibitors designed by molecular docking were significantly effective in inhibiting SecA ATPase in vitro [18]. Extensive use of antibiotics in open fields is not desirable due to environmental and human health concerns. Host-based treatments that modulate key genes involved in metabolic HLB syndrome are highly desirable. Data on citrus molecular responses to HLB can now be exploited to design small-molecule combinations to ameliorate the devastating symptoms. The aim of this study was to determine if small molecules are effective in modulating expression of key HLB-regulated or innate response genes after three to six days of treatment.

## Materials and Methods

### Plant material

#### Greenhouse trial

In 2011, Valencia orange scions on Kuharske Carrizo rootstocks were grown in one-gallon plastic nursery containers and kept in the greenhouse under natural light at 17 to 25°C. Graft inoculations were performed using a standard inverted “T” budding technique with C. Las-infected budwood tested as described [19]. Starting three months after budding, each plant was tested monthly using quantitative RT-PCR for C. Las species as described [19]. Each control or treatment was represented by nine to 10 trees. The control consisted of trees sprayed with distilled water. The first treatment consisted of a Silwet (0.12%), DKP3XTRA (32.5 mL/20 L) and LK-phite spray (2 mL/20 L). The other six spray treatments were composed of three different small-molecule combinations at two different concentrations each: 1) L-arginine at 1 mM or 0.5 mM, 2) 120 μM 6-benzyl adenine in combination with 15 or 30 μM gibberellin, and 3) 80 mM sucrose combined with the herbicide atrazine (2 μM or 1 μM). All seven treatments contained the surfactant Silwet and LK-phite at the same concentration used for the first treatment. Phenotypes were evaluated to determine any phytotoxic effects of these sprays. All treatments were sprayed on the citrus foliage; the volume sprayed per tree was enough to wet both upper and lower leaf surfaces just to the point of runoff. Gene expression analyses were conducted on RNA extracted three days following treatment. Three biological replicates of nine mature leaves harvested from three trees (three leaves per tree) were analyzed for each treatment. Collected leaves were immediately frozen in liquid nitrogen and kept at -80°C until RNA was extracted. Forty-two host genes were analyzed in mature symptomatic leaves of treated and untreated trees.

#### Field trial

The same treatments were applied during the field experiments, which were conducted in a commercial citrus orchard in Indian Ricer County, FL, composed of Valencia orange scions on Swingle rootstocks. The study was conducted on private land and the owner of the land gave permission to conduct the study on the site of our experiments. We also confirm that the field studies did not involve endangered or protected species. Twenty-four trees of medium height, 12 trees per row, were selected. These trees were mildly HLB-symptomatic and confirmed infected by C. Las through the same qPCR assay used for the greenhouse trial. The experimental design was a completely randomized blocks. There were eight treatments with three single-tree replicates. Samples were collected at three and six days following treatment. Each replicate was a pool of 10 mature symptomatic leaves per tree.

#### RNA extraction and qRT-PCR analysis

Total RNA was extracted from mature, fully-expanded leaves of plants grown in the greenhouse or orchard using the Rneasy Plant RNA Isolation kit (Qiagen Inc., Germany). The RNA concentration and purity were assessed by Nanodrop (Thermo Fischer Scientific Inc., MA, USA). RNA was stored at −80°C until analyzed. For each target gene, PCR primers were designed using Primer Express software (Applied Biosystems, Foster City, CA; S1 Table). DNase treatment and cDNA synthesis were completed following a combined protocol based on the Quantitect Reverse Transcription Kit (Qiagen Inc., Germany). A standard curve determined the linearity of amplicon quantity vs. initial cDNA quantity for each gene. Five μL cDNA at five ng/μL was diluted to a 12-μL final volume using Sybr Green Master Mix (Bio Rad Laboratories, Hercules, CA, USA). Amplifications were performed using standard conditions: 2 min at 50°C, 10 min at 95°C, 40 cycles of 15 s at 95°C, and 60 s at 60°C. Fluorescent signals were collected during the annealing temperature and ΔCT was calculated. Elongation factor 1 alpha (EF-1a, accession AY498567) was used as reference gene. ΔΔCT was determined by subtracting the average EF-1a CT from the average CT of the studied gene [9].

### Phenotypic measurements

Four phenotypic parameters for all treated and untreated trees were measured in the field: trunk diameter (mm), trunk height (m), width drill (m), and width row (m). These measurements were taken on 5/21/2014 and 6/28/2014 during the vegetative season.

### Statistical analysis

All statistical analyses of gene expression and phenotypic data were performed using SAS II (2008) SAS/STAT software (SAS Institute). Gene expression and phenotypic data were analyzed using ANOVA and a post-hoc test to identify significant differences among treatments. Principal component analysis (PCA) was used to reduce the dimensionality of the gene expression data. Data analysis was performed to alleviate possible bias caused by the collected material for each class or by other confounding factors. Principal component analysis was applied to the ratio matrix of gene expression data to examine the contribution of each target parameter to the separation of the sample classes. A biplot was constructed based on the first two principal components.

## Results

Gene expression analyses were conducted in the greenhouse and orchard. First, in greenhouse-grown plants, we quantified transcript abundance of 42 genes selected for their link with for being strongly up- or down-regulated by HLB syndrome [7,9] or for having a well-known role in plant responses to pathogen attacks [20]. Second, we selected a subset of five particularly representative genes to be analyzed in field-grown trees, to which we added an additional two genes previously linked with HLB syndrome in published data [5,9]. The presence of leaf drop or discoloration and other morphological features of trees were measured to check whether these treatments had deleterious effects on important vegetative parameters.

At advanced stages, HLB blocks sugar transport out of leaves, leading to starch accumulation in leaves, reduced photosynthesis and disrupted source-sink relationships [9]. Anatomical analysis showed that HLB caused phloem disruption, increased sucrose, and plugged sieve pores [6]. The disease also negatively modifies JA-SA crosstalk, leading to an ineffective innate immune response [7,9]. The three small-molecule treatments were selected for the potential to beneficially modulate these negative HLB-regulated responses. The combination of atrazine and sucrose upregulates genes associated with reactive-oxygen-species (ROS) defense mechanisms and sucrose metabolism [21,22]. We postulated that this treatment might upregulate genes that reduce sucrose and starch accumulation. Because L-arginine is the precursor of nitric oxide, which is involved in the SAR response and upregulates genes involved in secondary metabolism [23], we designed a second treatment consisting of two concentrations of L-arginine to induce upregulation of genes for secondary metabolic pathways such as phenols and terpenoids. Gibberellins boost systemic acquired resistance [20], favoring the resistance response against biotrophs such as C. Las [20]. Benzyladenine downregulates hexose transport in leaves, based on data deposited in the Genevestigator database. Indeed, we postulated that the combination of gybberellins and L-benzyladenine should have two synergistic effecs: 1) it should beneficially induce genes involved in the innate response against C. Las such as *WRKYs*, *MYC2* and salycilic acid methyl transferase, and 2) it should repress the expression of *GPT2* in symptomatic leaves, mitigating the deleterious HLB-driven upregulation [9].

### Greenhouse trial

#### Atrazine combined with sucrose treatment

The transcript abundance of genes related to carbohydrate metabolism varied significantly in response to atrazine combined with sucrose (Table 1).

**Table 1:** Relative transcript abundance of genes involved in carbohydrate metabolism.

Genes	Untreated	Control K-phite	1 μM Atrazine + sucrose	2 μM Atrazine + sucrose	120 μM BA + 30 μM GA	120 μM BA + 15 μM GA	1 mM Arginine	0.5 mM Arginine
Starch metabolism								
Alpha- amylase	0.470 b	4.950 ab	3.320 ab	4.860 ab	87.140 a	5.210 ab	8.520 ab	3.880 ab
Water dikinase starch degrad.	0.317 d	0.087 d	0.063 d	1.096 a	0.185 d	0.732 b	0.635 bc	0.364 cd
GPT2	0.569 a	0.012 b	0.219 b	0.171 b	0.025 b	0.107 b	0.243 b	0.119 b
Starch synthase	0.672 b	2.429 ab	4.256 a	1.886 ab	0.792 b	2.870 ab	2.016 ab	3.029 ab
Sucrose metabolism								
Invertase	0.839 a	0.766 a	0.351 a	0.274 a	0.296 a	0.851 a	0.758 a	0.611 a
Sugar signaling	0.546 a	2.116 a	0.937 a	2.769 a	7.359 a	1.763 a	1.750 a	3.209 a
Susy	2.310 b	4.218 ab	5.573 a	2.131 b	2.795 b	3.412 ab	4.296 ab	2.022 b
Sps	0.729 b	2.023 b	5.700 b	13.486 a	1.031 b	3.393 b	2.637 b	5.966 b

Means of three replicates were indicated. Letters means significant differences using ANOVA (P < = 0.05) and post-hoc test.

The *sucrose synthase* (Susy) and starch synthase transcripts were more abundant in 1 μM atrazine-treated plants. *Sucrose-phosphate-synthase* and *water dikinase starch degradation* (*GW*D) gene were upregulated by 2 μM atrazine + sucrose. Taken together, these findings highlight that activation of *sucrose synthase* should counter the accumulation of sucrose in symptomatic leaves, while the upregulation of *GWD* should promote degradation of accumulated starch.

Defense responses and hormone-related genes were affected by atrazine + sucrose treatments. *WRKY33* was upregulated by 1 μM atrazine + sucrose (Table 2).

**Table 2:** Relative transcript abundance of genes involved in plant innate immune responses.

Genes	Untreated	Control K-phite	1 μM Atrazine + sucrose	2 μM Atrazine + sucrose	120 μM BA + 30 μM GA	120 μM BA + 15 μM GA	1 mM Arginine	0.5 mM Arginine
RAD51 D	0.197 b	0.175 b	0.690 ab	1.878 a	0.335 b	0.350 b	0.272 b	1.362 ab
BZIP45	5.781 ab	7.650 ab	10.216 ab	11.644 ab	13.370 a	5.197 a	8.997 ab	4.983 ab
TGA5	2.391 b	3.107 ab	4.370 ab	3.366 ab	2.203 b	3.026 ab	6.010 a	2.201 b
RGA1	3.537 a	4.092 a	5.953 a	5.301 a	5.540 a	4.968 a	3.562 a	13.320 a
WRKY33	1.752 b	1.099 b	6.372 a	2.891 b	2.425 b	1.128 b	1.510 b	1.074 b
WRKY48	8.133 ab	4.783 b	6.387 ab	13.873 a	10.300 ab	3.918 b	3.405 b	1.928 b
WRKY54	1.701 a	4.708 a	4.753 a	6.452 a	3.576 a	6.850 a	2.723 a	1.808 a
EDS1	1.491 a	1.905 a	11.022 a	9.360 a	6.914 a	8.289 a	6.561 a	4.319 a
ERF1	7.064 b	6.616 b	17.691 ab	29.435 a	18.566 ab	9.641 b	5.636 b	11.654 b
MYC2	5.369 cd	3.872 d	10.282 abcd	6.505 bcd	17.029 ab	18.585 a	4.134 d	6.320 bcd
PR1	0.053 b	0.229 ab	0.078 b	0.970 ab	0.454 ab	0.119 ab	0.832 ab	2.27 ab
SR1	1.670 a	2.690 a	6.680 a	4.720 a	46.630 a	5.590 a	6.950 a	5.210 a
WRKY59	1.376 b	1.653 b	1.709 b	2.632 b	8.332 a	1.366b	2.996 ab	1.035 b

Means of three replicates were indicated. Letters means significant differences using ANOVA (P < = 0.05) and post-hoc test.

A *zinc ion binding transcription factor*, *heat shock protein 82* (*HSP82*) and *ERF1* were strongly induced by 2 μM atrazine (Tables 2 and 3).

**Table 3:** Relative transcript abundance of genes involved in stress responses and secondary metabolism.

Genes	Untreated	Control K-phite	1 μM Atrazine + sucrose	2 μM Atrazine + sucrose	120 μM BA + 30 μM GA	120 μM BA + 15 μM GA	1 mM Arginine	0.5 mM Arginine
Stress-related and cell wall								
HSP21	23.350 c	308.390 a	24.110 c	106.950 bc	27.670 c	83.290 bc	97.860 bc	288.346 a
HSP82	1.688 c	5.208 ab	0.729 c	5.585 a	3.364 abc	5.448 ab	4.332 ab	0.920 c
ABC Transporter	2.189 de	1.110 de	0.537 e	15.798 b	6.580 cd	24.822 a	9.245 c	1.229 de
ATPtranslocase2	0.022 a	0.052 a	0.032 a	11.499 a	0.012 a	0.148 a	0.125 a	0.029 a
Sulfotransfer. 1	0.298 c	0.008 c	0.6987 c	0.6015 c	2.678 b	6.932 a	0.483 c	0.136 c
NNLTP	0.022 a	0.052 a	0.032 a	11.499 a	0.012 a	0.148 a	0.125 a	0.029 a
PSBW	2.394 c	3.178 c	8.020 b	6.470 bc	2.287 c	6.226 bc	2.539 c	12.839 a
Pectate lyase 5	0.427 b	0.242 b	0.169 b	0.919 ab	0.231 b	4.233 a	0.817 ab	0.589 ab
Secondary metabolism								
Terpene synthase 14	0.198 b	0.184 b	0.402 b	0.112 b	0.101 b	0.345 b	0.051 b	1.944 a
Terpene synthase 21	3.472 a	1.309 a	1.026 a	5.870 a	4.891 a	1.849 a	1.543 a	6.564 a
Terpene synthase 3	0.76 a	0.53 a	4.92 a	38.40 a	0.64 a	1.37 a	0.32 a	0.19 a
B-Amyrin	1.457 b	2.300 ab	2.178 b	3.504 ab	22.614 a	9.651 ab	3.253 ab	6.649 ab

Means of three replicates were indicated. Letters means significant differences using ANOVA (P < = 0.05) and post-hoc test.

*JIN1* was induced by 1 μM atrazine + sucrose (Table 4).

**Table 4:** Relative transcript abundance of genes involved in hormone-related pathways.

Genes	Untreated	Control K-phite	1 μM Atrazine + sucrose	2 μM Atrazine + sucrose	120 μM BA + 30 μM GA	120 μM BA + 15 μM GA	1 mM Arginine	0.5 mM Arginine
Gibberellins								
GA2-oxidase	0.395 a	0.676 a	0.556 a	5.276 a	2.718 a	1.752 a	0.199 a	1.379 a
Gibberelin-2-oxygenase	0.765 a	0.357 a	0.793 a	1.367 a	2.629 a	3.965 a	1.158 a	0.487 a
Auxins and Benzyl-adenine								
GH3.1	0.077 b	0.021 b	0.135 b	0.511 a	0.048 b	0.070 b	0.118 b	0.255 ab
Ka02	2.837 ab	3.661 a	2.532 ab	3.608 a	0.139 b	4.164 a	0.929 ab	2.794 ab
Jasmonic acid								
12-oxophytodi. reductase 1-like	0.444 b	0.290 b	0.322 b	1.986 a	0.174 b	1.063 ab	1.964 a	0.732 ab
Salicylic acid methyl transferase								
SA-methyl transferase	0.033 b	0.015 b	0.052 b	0.277 a	0.314 a	0.091 a	0.360 a	0.434 b
Ethylene								
ACS-1	3.960 a	3.580 a	5.039 a	6.202 a	2.025 a	1.723 a	4.595 a	4.247 a

Means of three replicates were indicated. Letters means significant differences using ANOVA (P < = 0.05) and post-hoc test.

*Salicylic acid methyl transferase* and *12-oxophytodienoate reductase 1-like* were upregulated by 2 μM atrazine + sucrose.

#### Gibberellin and benzyl-adenine treatment

GA + BA treatments were tested to determine their effects on key genes involved in plant innate immune responses and carbohydrate metabolism. Among carbohydrate metabolism genes, alpha-amylase was significantly induced by 120 μM gibberellins combined with 30 μM benzyl-adenine (Table 1). Among innate immune response genes, *WRKY54* was upregulated in response to 120 μM gibberellins + 15 μM benzyl-adenine while *WRKY59* was enhanced by 120 μM gibberellins + 30 μM benzyl-adenine (Table 2). *Sulfotransferase1* was enhanced by both gibberellin and benzyl-adenine treatments. *MYC2* was upregulated by benzyladenine + gibberellin.

Among the secondary metabolism and stress response genes, 120 μM gibberellins + 15 μM benzyl-adenine enhanced expression of *HSP82* and two genes encoding *pectate lyases* involved in cell wall metabolism (Table 3). *β-amyrin* was enhanced by 30 μM gibberellins combined with benzyl-adenine.

BA + GA treatments induced *SA methyl transferase* (Table 4). BA + GA partially induced HLB-related changes to SA-mediated defense response, but *EDS1* was not altered by the treatment, so the plant probably still can’t downregulate jasmonic antagonistic signaling. *Ka02* involved in cytokinin metabolism was higher in response to 15 μM than to 30 μM benzyl-adenine.

#### Arginine treatment and K-phite treatments

0.5 mM L-arginine upregulated *HSP21* and *terpene synthase3* (Table 3). Arginine treatments did not alter the expression of *terpene synthase14* and *terpene synthase21*, so the treated plant is still unable produce important terpene compounds. *TGA5* and *HSP82* were enhanced by 1 mM L-arginine. K-phite treatment repressed *glucose-phosphate-transporter2* (*GPT2*). This downregulation may have a beneficial effect since this gene allows glucose import into the chloroplast and starch accumulation. K-phite significantly induced *HSP21*, a chaperone involved in functional protein stability.

#### Principal component analysis

**Fig 1:**
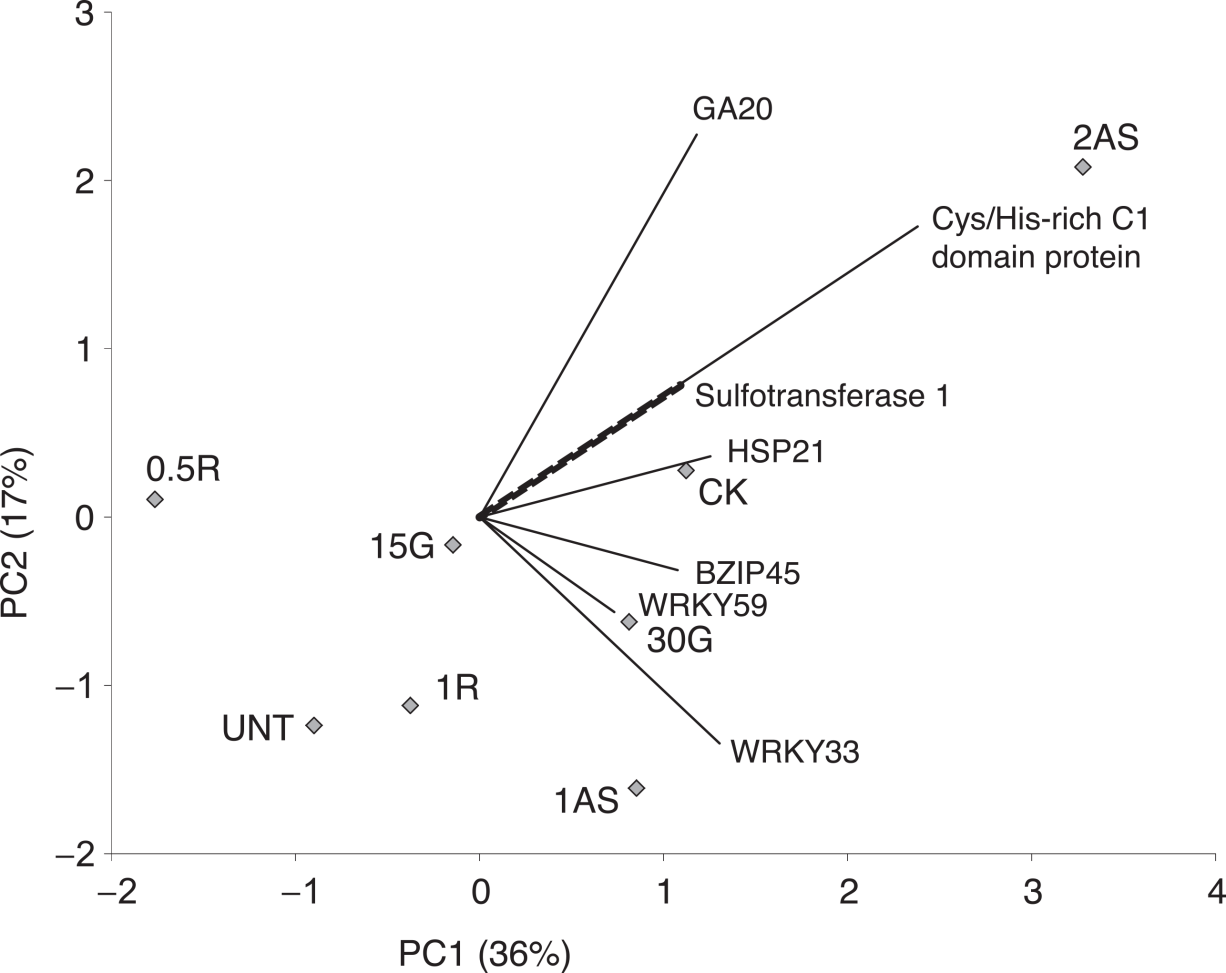
Overall analysis of HLB-regulated changes in carbohydrate metabolism. Principal component analysis of treated and untreated Citrus categories in relation to genes involved in sucrose and starch pathways. UNT = Untreated with hormones (Control), CK = treated with K-phite, 30G = 30 μM gibberellin, 15G = 15 μM gibberellin, 1R = 1 mM L-arginine, 0.5R = 0.5 mM L-arginine, 2AS = sucrose combined with 2 μM atrazine, 1AS = sucrose combined with 2 μM atrazine. SPS = sucrose-phosphate-synthase.

**Fig 2:**
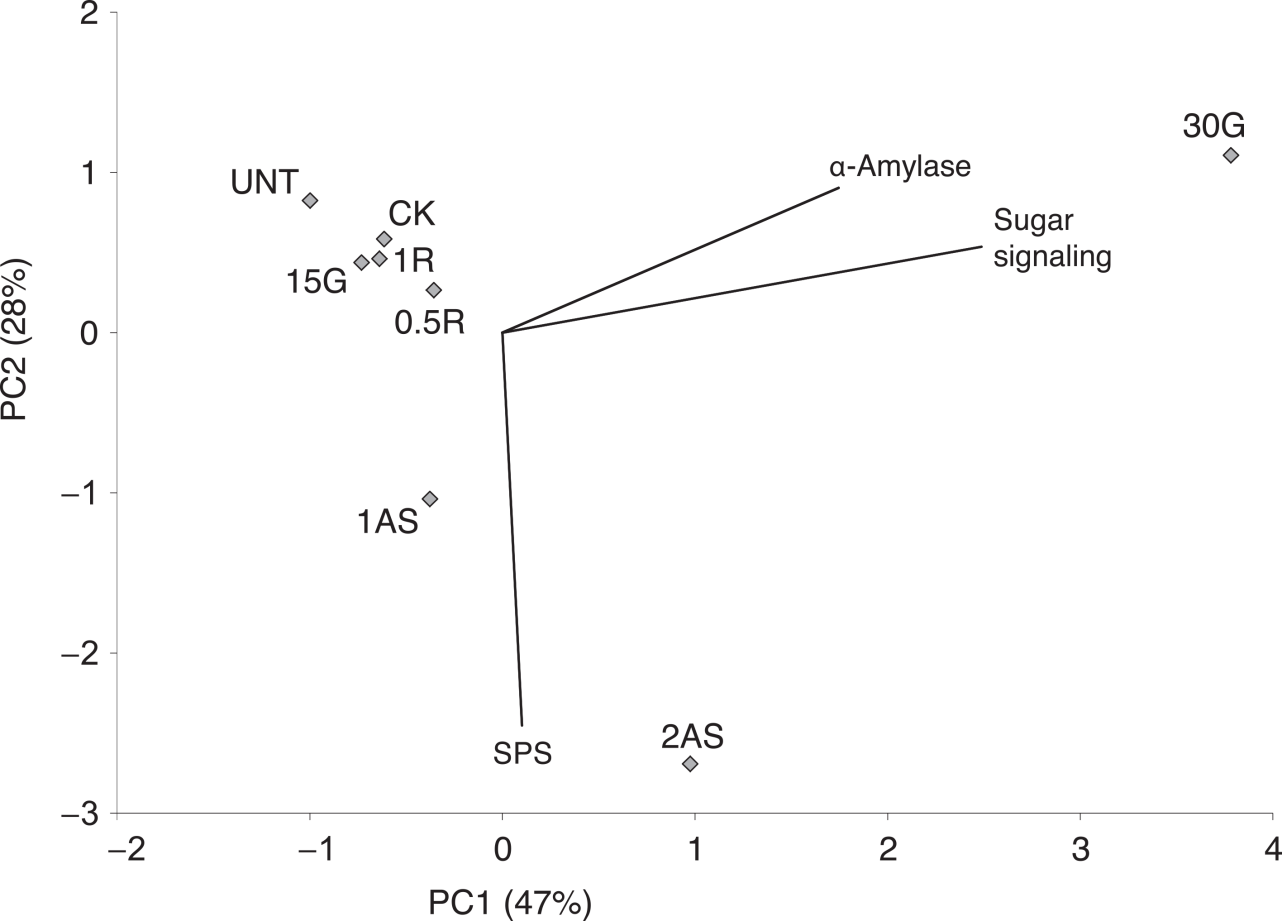
Overall analysis of HLB-regulated changes in biotic stress response. Principal component analysis of treated and untreated Citrus categories in relation to genes involved in biotic stress responses. UNT = Untreated with hormones (Control), CK = treated with K-phite, 30G = 30 μM gibberellin, 15G = 15 μM gibberellin, 1R = 1 mM L-arginine, 0.5R = 0.5 mM L-arginine, 2AS = sucrose combined with 2 μM atrazine, 1AS = sucrose combined with 2 μM atrazine. GA20 = Ga2-oxidase, HSP21 = Heat shock protein 21.

Two principal component analyses were conducted to independently assess two gene subsets: 1) genes involved in sucrose and starch metabolism (PCA-1; Figs 1 and 2) genes involved in hormone-related proteins and biotic stress responses (PCA-2; Fig 2).

In PCA-1, the first two principle components explained 47 and 28% of data variability, respectively. The 2 μM atrazine + sucrose treatment separated from the rest of the treatments. *SPS* greatly contributed to this separation. 120 μM BA + 30 μM GA was also highly discriminated from the rest of the treatments. The other treatments were not distinct from the untreated controls.

PC 1 and PC 2 of PCA-2 (Fig 2) explained 36 and 17% of data variability, respectively. The 1 mM L-arginine treatment was not distinct from untreated conditions, but the 2 μM atrazine + sucrose treatment was highly distant. *GA2-oxidase*, *zinc ion binding*, and *cysteine-histine rich domain C1* gene contributed significantly to the separation of 2 μM atrazine + sucrose treatment. *WRKY33* highly contributed to the separation of the 1 μM atrazine + sucrose treatment.

### Field trial

Small-molecule regulating treatments investigated in the greenhouse were also applied to HLB-symptomatic trees in a young orchard where disease symptoms were frequently present. The aim of this trial was to determine whether the same treatments used in the greenhouse could modulate the expression of key host genes at three and six days after their application. Seven key genes were selected to monitor the transcriptomic regulation of the treatments for two reasons: 1) previous data found them highly characteristic of an HLB-induced response [7,9] and 2) they play a key role in innate immune responses. A gibberellin responsive gene was used as a marker to determine the efficacy of GA + BA treatments to modulate gene expression under field conditions. *GPT2* is a key HLB-regulated gene involved in glucose import into the chloroplast and is linked to the increased accumulation of starch in symptomatic leaves. The other genes were involved in plant defense and hormonal-mediated innate responses. *WRKY70* and *EDS1* are key points of regulation of JA-SA crosstalk. *PR1* upregulation is a beneficial against C. Las since this gene is involved in the systemic acquired resistance response. *WRKY48* and *WRKY54* were induced by HLB in previous studies [7,9].

**Fig 3:**
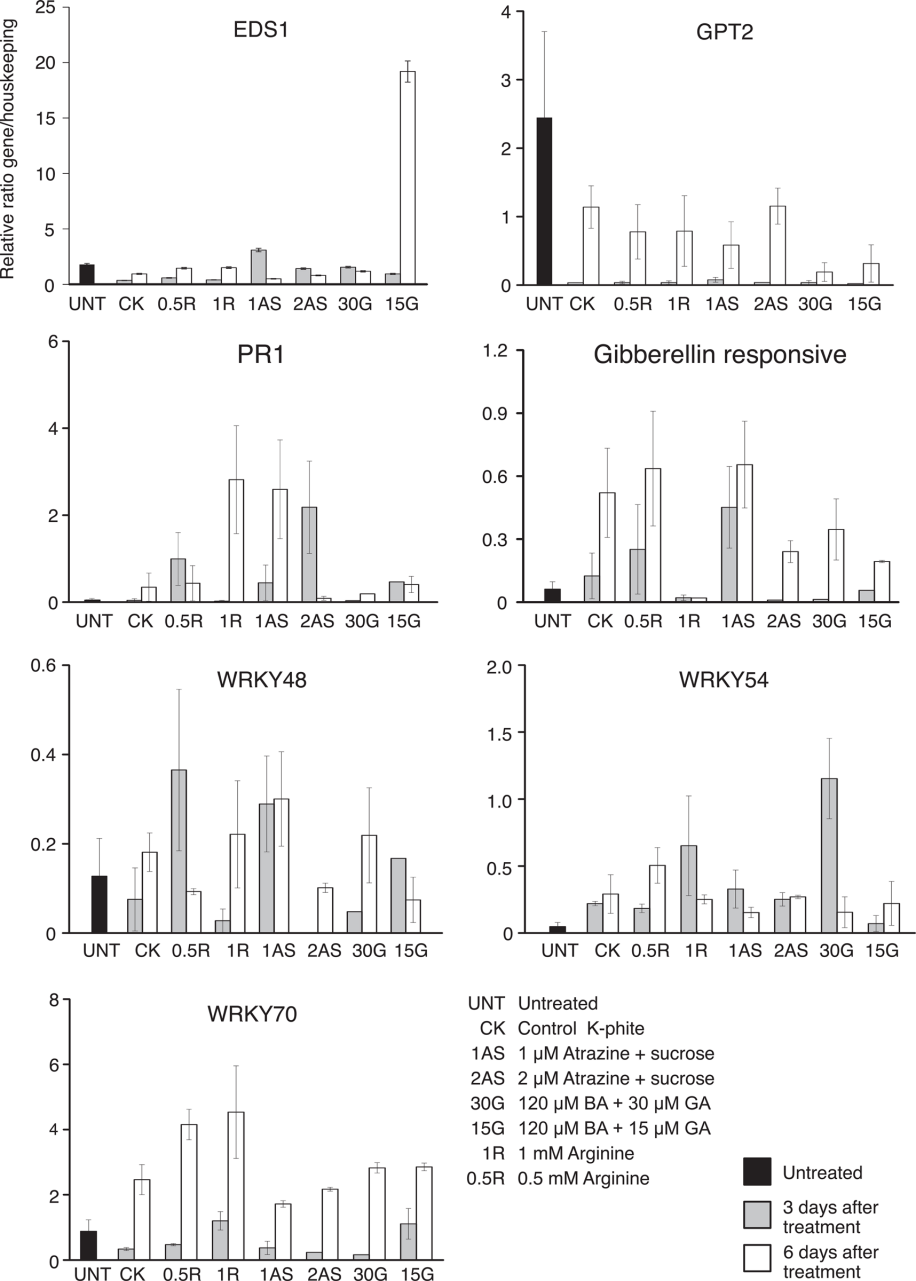
Expression of seven host genes in response to spray treatments in field conditions. Relative expression of each gene and treatment was shown as average of three biological replicates. Standard deviations were indicated.

At three days after treatment, 1 μM atrazine + sucrose induced the gibberellin-responsive protein and *PR1* and repressed *WRKY48* (Fig 3).

In addition, 30 μM GA + 120 μM BA induced *WRKY48* and *WRKY54*. This finding may have positive effects on infected Citrus since the two genes are involved in salicylic acid-mediated responses against biotrophs. 0.5 mM L-Arginine upregulated *WRKY48* and *EDS1*. *WRKY70* was enhanced by 1 mM arginine and 15 μM GA combined with 120 μM BA.

At six days following treatment, some important changes in expression of the seven host biomarkers were observed. 1 μM atrazine upregulated *PR1*. 2 μM atrazine and 0.5 mM L-arginine repressed *WRKY48*. A general inhibition of *GPT2* was observed in all treated trees at both three and six days after treatment. This repression was particularly evident in leaves sprayed with gibberellins + benzyladenine.

#### Phenotypic measurements and pathogen qPCR detection

No obvious symptoms of a harmful spray as leaf drop or discoloration was observed in treated trees. The tree trunk diameter, height, width drill, and width row of the treated trees grown under field conditions were measured in May and June 2014 (Table 5).

**Table 5:** Phenotypic measurements in response to the seven treatments and control (untreated).

1st Measurement (5.21.14)	Untreated	120 μM BA + 30 μM GA	120 GA + 15 μM BA	1 μM Atrazine	2 μM Atrazine	.12% Siluet K-Phite +	0.5 mM L-Arginine	1.0 mM L-Arginine
Trunk Diameter (mm)	90.770 a	91.537 a	82.280 a	96.097 a	88.197 a	83.827 a	96.427 a	98.747 a
Tree Height (m)	2.9533 ab	3.0000 ab	2.5533 b	3.1667 ab	3.1900 ab	2.8133 ab	3.2567 ab	3.3200 a
Width Drill (m)	2.4167 a	2.7400 a	2.4733 a	2.7000 a	2.6767 a	2.4433 a	2.7467 a	2.6233 a
Width Row (m)	2.4767 a	2.7667 a	2.4400 a	2.7400 a	2.5267 a	2.5533 a	2.7400 a	2.6800 a
2nd Measurement (6.28.13)	Untreated	120 μM BA + 30 μM GA	120 GA + 15 μM BA	1 μM Atrazine	2 μM Atrazine	.12% Siluet K-Phite +	0.5 mM L-Arginine	1.0 mM L-Arginine
Trunk Diameter (mm)	70.940 a	77.427 a	70.503 a	80.813 a	75.207 a	72.513 a	82.293 a	83.617 a
Tree Height (m)	2.5500 abc	2.6833 ab	2.3533 bc	2.7067 ab	2.6733 abc	2.3100 c	2.7267 a	2.7700 a
Width Drill (m)	2.1767 a	2.5400 a	2.3133 a	2.4233 a	2.4000 a	2.4200 a	2.6533 a	2.4400 a
Width Row (m)	2.0700 c	2.6167 ab	2.2733 bc	2.4900 abc	2.3033 abc	2.2667 bc	2.7267 a	2.4267 abc

Means of three replicates were indicated. Letters means significant differences using ANOVA (P < = 0.05) and post-hoc test.

The aim of this analysis was to determine whether treatments were detrimental to tree growth or had undesirable phenotypic effects. No significant phenotypic differences were observed among untreated and treated trees except width row, which was significantly lower in untreated trees than in trees treated with 30 μM GA + 120 μM BA. There were no visible discolorations or other signs of plant distress from the spraying. No set of trees had visibly different vegetative vigor.

The quantification of pathogen titer was performed after three months from treatments as previously indicated to check if it was not changed in response to treatments.

## Discussion

Our objective was to test the ability of six combinations of small-molecule compounds to modulate expression of key genes involved in HLB syndrome and innate immune responses shortly after treatment. We did not pretend to reduce pathogen titers or cure the plants with only one treatment. Before performing a long-term study, we wanted to evaluate the ability of the treatments to modulate expression of a subset of key genes that are altered during HLB syndrome. As we expected pathogen titers did not significantly differ among treated and control trees. A long-term study will reveal if repetitive and continuous applications will reduce pathogen concentrations and symptom severity.

Treatments were designed based on previously proposed hypotheses. Sucrose-induced protection against atrazine effects was linked to upregulation of reactive oxygen species (ROS) defence and repair mechanisms [21].

Nitric oxide (NO) is produced by l-arginine. Treatment with arginine provoked resistance against *Botrytis cinerea* in tomato at three to six days after treatment. Endogenous NO concentrations correlated positively with induction of key enzymes involved in biotic stress responses such as phenylalanine ammonia-lyase, chitinase, β-1,3-glucanase and polyphenoloxidase [23].

Gibberellins regulate plant growth by modulating degradation of growth-repressing DELLA proteins that promote susceptibility to biotrophic pathogens and resistance to necrotrophic pathogens [24]. This is accomplished by modulating the relative strength of the SA and JA signaling pathways [24]. Through regulation of DELLA stability, gibberellins affect the SA-JA-ET network and plant immune response. Genevestigator showed that benzyl-adenine downregulated the glucose-phosphate transporter in *Arabidopsis*. Since this gene is induced by HLB metabolic syndrome [5,9], benzyl-adenine treatments might help mitigate the negative effects of HLB on leaf metabolism. In combination, the two hormones may beneficially modulate key HLB-regulated genes involved in carbohydrate metabolism [7,9].

K-phite mineral solution was also tested, alone or in combination with the three small molecule compounds. This treatment was considered because of contrasting published reports on the effects of nutrient solutions such as K-phite [25]. Mineral solutions increased the concentrations of important N, Mn, Zn and B ions in leaves and long-term application reduced pathogen titer, leaf size, and leaf weight [25]. Although enhanced nutritional solutions composed of essential micronutrients did not improve fruit production and quality of C. Las-infected trees [26], others results support the hypothesis that the pathogen severely affects nutrient patterns [27]. In addition, foliar nutrition and soil conditioners helped reduce economic and production losses due to HLB [28,29].

To determine how the treatments affected the metabolism of infected trees, 42 genes were selected from previously published Citrus transcriptome data [7,9]. These genes fell into three subsets involved in: 1) carbohydrate metabolism and signaling, 2) innate immune responses, including key players in JA-SA signaling, crosstalk and induced responses, or 3) other genes involved in biotic stress responses such as those involved in hormone-related pathways, secondary metabolism and stress-preventing factors. From these biomarkers, we chose seven representative biomarkers to be followed under field conditions in response to the same treatments. Treated plants were tested for the presence of C. Las using qPCR and showed clear HLB symptoms.

QRT-PCR analyses were conducted at three to six days after treatment. As expected, we observed no significant changes in pathogen titer. Repeated applications of treatments (at least weekly) should eventually affect the titer. Since we only treated infected trees once, an analysis of pathogen titer after treaments was outside the scope of this study. Long-term studies on the effects of repeated applications of these treatments should test pathogen titer using qPCR.

### Atrazine combined with sucrose

The first small-molecule treatment tested was the combination of sucrose and the herbicide atrazine. Atrazine is a well-known photosystem II inhibitor that affects plant gene expression, seedling physiology, and potentiality impairs protein translation and the ROS defense mechanism [30]. However, in combination with sucrose, atrazine induces xenobiotic and ROS signaling. In addition, this treatment upregulated important classes of antioxidant enzymes [21,22]. These findings were consistent with our unpublished findings that *glutathione-S-transferases* are upregulated in more tolerant Citrus genotypes.

Here we observed that atrazine combined with sucrose drastically affected some key genes responsible for HLB-induced carbohydrate changes. Increased sucrose concentrations have been found in in C. Las-infected leaves [6,11]. 1 μM atrazine + sucrose enhanced sucrose synthase while 2 μM atrazine + sucrose upregulated the water dikinase starch degradation gene and sucrose-phosphate-synthase. Atrazine upregulated *alpha-amylase*, which was repressed in mature HLB-infected Citrus leaves [9] but upregulated in infected Citrus stems [31]. Taken together, these findings lead us to speculate that atrazine + sucrose might help sucrose degradation by activating *sucrose synthase*. In addition, upregulation of *alpha-amylase* may increase starch degradation in HLB-infected plants where its accumulation is advanced.

Atrazine (1 μM) combined with sucrose upregulated *WRKY33*. *Brassica napus* plants overexpressing *BnWRKY33* had increased resistance to *Sclerotinia sclerotiorum* infection [32]. This effect was mediated by SA [32]. *WRKY33* upregulation allowed resistance to the necrotroph *Botrytis cinerea* in *Arabidopsis* [33]. Loss of *WRKY33* function induces salicylic acid (SA)-mediated responses, increases salicylic acid and represses jasmonic acid (JA)-mediated responses [34].

Overall, our results support the hypothesis that this treatment could beneficially modulate key HLB-regulated genes associated with the well-known HLB carbohydrate metabolic syndrome [9]. The changes to expression of some key genes involved in sugar and starch metabolism could beneficially modulate the metabolic responses of HLB disease in photosynthesizing Citrus leaves, restoring a more normal source-sink relationship and potentially inhibiting the characteristic and deleterious syndrome in the fruit.

### Gibberelllins combined with benzyl adenine treatments

A mixture of gibberellin (GA3) and 6-benzyladenine (BA) was tested to modulate jasmonic acid-salicylic acid (JA-SA) crosstalk in favor of responses to biotrophs such as C. Las. Our hypothesis was that gibberellin treatments may activate SAR responses through positive regulation of hormone-mediated crosstalk regulating biotic stress responses [19]. Some key genes in hormone-related pathways and JA-SA crosstalk were chosen as indicators of treatment effects.

*MYC2* was significantly induced by both 15 and 30 μM gibberellin treatments. *MYC2* is a transcription factor composed of a basic helix-loop-helix (bHLH) domain that activates and represses specific JA-responsive gene expression in *Arabidopsis* [35]. *MYC2* also induced responses to abiotic stress mediated by abscissic acid in Arabidopsis [36] and suppressed salicylic acid-mediated responses in Arabidopsis [37]. Upregulation of *WRKY54* in gibberellin-treated field trees is also interesting because this gene is a positive regulator of resistance against *Erwinia amylovora*, the agent of fire blight in the Rosaceae family.

The plant immune regulator *EDS1* (Enhanced Disease Susceptibility1) plays a fundamental role in resistance mechanisms to biotrophs and hemi-biotrophs [38,39]. This role is due to the formation of complexes with *PAD4* and *SAD101* in both cytoplasm and nucleus [40]. The 15 μM GA + BA treatment enhanced EDS1 at six days after treatment in the field. The increase in *EDS1* transcripts after application of these small molecules could help activate SAR response against pathogen infections. Mutant screening showed that upregulation of *EDS1* induces non-host resistance against *E*. *amylovora* in *Arabidopsis* by activating *WRKY46* and *WRKY54* genes [41].

Our hypothesis was partially confirmed. GA + BA may beneficially increase innate responses by inducing *EDS1* and *MYC2*. Although long-term field trials are required, we speculate that continuous application of this small molecule mixture could stimulate improved SA-JA crosstalk

### L-arginine treatments

The third small-molecule treatment was composed of L-arginine, used in two concentrations. L-arginine positively regulates key genes involved in innate immune responses [23]. L-arginine may act on nitric oxide and directly upregulate key genes in salicylic acid signaling. Increased endogenous NO concentrations after L-arginine treatment in pre-harvest tomatoes correlated positively with increased defensive enzyme activity and postharvest disease resistance [15]. *PR1*, *WRKY70* and *WRKY54* were upregulated by 1 mM L-arginine under field conditions. *WRKY* transcription factors are important regulators of responses to abiotic and biotic stresses. *WRKY54* and *WRKY70* play a key role in a regulatory network that affects leaf senescence by interacting with another WRKY factor [42].

In our field trial, arginine induced some key important gene regulation that should benefit SAR responses.

### Common effects among treatments

Glucose accumulation induced by C. Las infection is transported to the plastid by hexose transporters [5,9,43]. GPT2 is a key player in HLB-mediated starch accumulation in leaves because this gene mediates glucose import into the chloroplast in infected leaves [5,9]. *GPT2* was significantly repressed by all spray applications at both three and six days after treatment under field conditions. This inhibition may reduce the amount of glucose carried into the plastid and thus starch accumulation, with consequent improvement of disrupted source-sink relationships.

**Fig 4:**
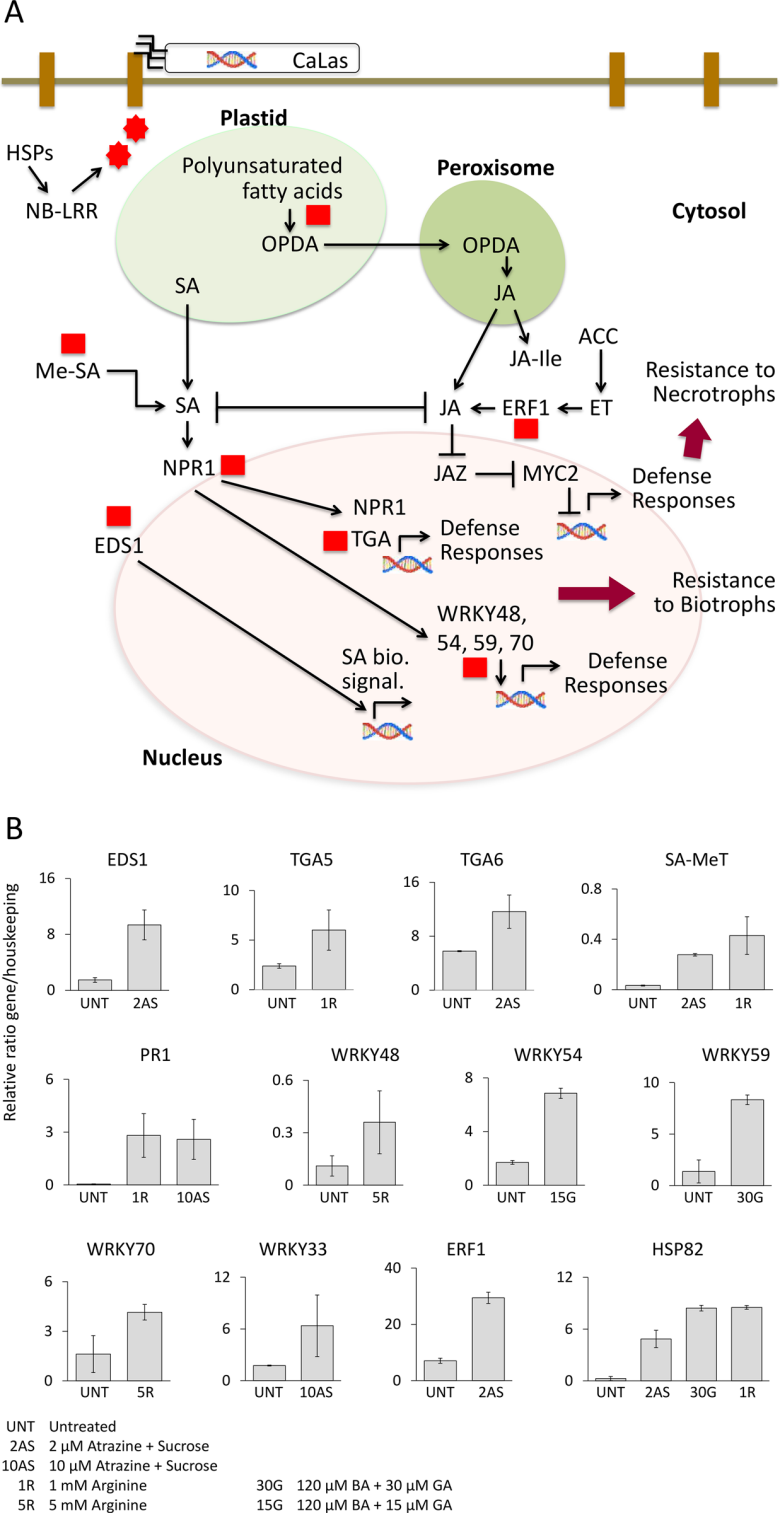
Key differentially regulated genes in response to treatments involved in biotic stress responses.

Gene expression changes observed in this work corroborated the hypothesis that these spray treatments may help stimulate systemic acquired resistance responses by activating key genes involved in innate responses (Fig 4).

PR gene induction is mediated through interaction with TGA transcription factors [15]. The observed upregulation of *TGA5* and *TGA6* by L-arginine and 2 μM atrazine + sucrose, respectively, might help stimulate defense responses against C. Las infection.

Arginine and atrazine sprays upregulated the *PR1* gene under field conditions. PR1 protein is the hallmark of the defense response induction mediated by salicylic acid through systemic acquired resistance [44]. Molecular action of this protein against pathogens is still unclear, although antifungal properties have been attributed to it [45]. *PR1* also interacted with fungal toxin activities, mediating necrosis in sensitive wheat [31]. This gene was not activated in response to C. Las infections in orchard trees [9].

Interestingly, all three treatments upregulated *HSP82*. Previous data on C. Las-infected citrus leaves and fruits showed that C. Las caused a significant repression of genes encoding chaperones [7–9]. Modified expression of these genes plays a key role in general stress conditions [46, 47]. A link between reduced HSP protein amount and HLB symptoms was also confirmed by analysis [10].

## Conclusions

Present data confirmed our hypothesis that these small-molecule sprays may affect transcript abundance of key genes involved in HLB carbohydrate metabolic syndrome and innate responses. As expected, there were no phenotypic changes in response to treatments at one to two months after treatment. Treatment sprays did not cause negative effects such as leaf drop or discoloration. As expected, tree measurements showed almost no differences between treated and untreated trees in field conditions. We believe that beneficial effects are likely to be seen only if treatments are applied frequently before or at the onset of visible HLB symptoms. Here, our aim was to analyze the molecular effects of these treatments on gene expression several days after treatment. Future studies should examine long-term molecular and phenotypic improvements associated with ongoing applications to young trees infected with C. Las.

## Supporting Information

S1 Table List of primers used for each gene analyzed using qRT-PCR. (DOCX)
