# Supplementary material for: Molecular Responses to Small Regulating Molecules against Huanglongbing Disease
Source: PLoS One. 2016 Jul 26;11(7):e0159610. doi: 10.1371/journal.pone.0159610 (PMC4961454; doi:10.1371/journal.pone.0159610)
Supplement: S1 Table — (DOCX) [file pone.0159610.s001.docx]

**Table S1.** List of primers used for each gene analyzed using qRT-PCR.

| **GENES** | **ACCESSION NUMBER** | **SEQUENCES** |
| --- | --- | --- |
| RAD51 D | XM_015533367 | F: CCAGCACATTATGGAGGCTT  R: CCAGCACATTATGGAGGCTT |
| PECTATE LYASE 5 | XM_006483937.2 | F: GCAAGTCACGATTGCTTTCA  R: CGCATACATCTCCCAATGTG |
| BZIP45 | XM_006432367 | F: GTTGGAGCCTTTGACAGAGC  R: ATAGTTTGCCACGTTCCCAG |
| WRKY70 | XM_006481140 | F: TGAAAGCAGCAAGAGCTCAA  R: TTTTGCCCATATTTCCTCCA |
| WRKY48 | XM_006488761 | F: TCATCATCAAACGAAGCAGC  R: CTCTTTGTCATGAACGCGAA |
| EDS1 (Enhanced Disease Susceptibility 1) | XM_006494235.2 | F: TGAGGCGAGAAAGTTGGTCT  R: TTGGGTTCAAGAAATCGAGG |
| ETHYLENE RESPONSIVE TRANSCRIPTION FACTOR 5 | XM_006467633 | F: TCGCGGCTGAGATTCGA  R: AACGTGCCCAGCCATATCC |
| ATP TRANSLOCASE | XM_006465343.2 | F: CAAGGGTTTGTCCCAAAGGA  R: GGCTGCCCATCAGCAGAA |
| GA2-oxidase | LC149864 | F: GTCCGAAGCTTTTCACATTCCT  R:GAGTAGTGAAAGCAGCAGCAGAAG |
| GH3.1 | XM_006486899.2 | F: TGCCAACGGCGATCGT  R: CTGGTGAGGAATTCGGAGATG |
| ALPHA- AMYLASE | XM_006473264 | F: GCCATTCCCATCGGATAAAGT  R: GGTGCCGGGATGTGTGA |
| WATER DIKINASE STARCH DEGRADATION | XM_015525686 | F: AGGACTTGGCTGGAATGTCA  R: TGGTCATGATCTGTTGGGCT |
| GPT2 | XM_006467936 | F: AACTTCGTGTGGTGGGTAGC  R: CCAATGCTGAATGTCAATGG |
| GIBBERELLIN-2-OXYGENASE | XM_006436699.1 | F: CAGCTCATTTTGCCAGGAAGT  R: ACAACAACACACTCACGGATATTTC |
| HSP21 (Heat Shock Protein) | XM_006447117 | F: ACGCCTTGGGCCATCA  R: TGCCAGGCACGTCTAACCTAAT |
| INVERTASE | XM_006488730.2 | F: CGATGGAACACTTGAAGAGGTTT  R: GGGCAACACGACCAATGG |
| B-AMYRIN | XM_006485364.2 | F: TGGCAGATTGGGAGAGAAGAA  R: GCAAAGGCCAGCCCATT |
| SA-METHYLSALICYLATE | XM_006466773 | F: TCCAATGGCTATCTCAAGTTCCA  R: GGCCATGAAAATGTTCCCTTT |
| MYC2 | XM_015534207 | F: ACCAGAGTGGTGGGCTTATG  R: ATGGCCCTCTTTCTCTCCAT |
| NNLTP (lipid transfer pritein) | XM_006481120 | F: GCCAGTGTGGCGTCAACA  R: CATCTCACTCTTGCTGCAGTCAA |
| PR1 (Pathogenesis-related 1) | XM_006486756 | F: TGTATGGATGGGAAGCCATT  R: TACCTCGGGGATCGTAGTTG |
| PSBW ( photosystem II reaction center W protein) | XM_006487724 | F:GATGAGAGAATGACCACAGAAGGA  R: CCACCCAAGAGTGTTGTGCTT |
| RGA 1 (DELLA PROTEIN) | XM_006469132 | F: GGACCGGTTCACAGAGTCAT  R: GCTTGCTTGTACGCATTTGA |
| SPS (Sucrose-phosphate -synthase) | XM_006478278 | F: GGACGGCACAGGCAGTTG  R: TCGCGTGCGCCACAT |
| SR1 (Signal responsive 1) | XR_370867 | F: AGCAAGTCTCCAAAAGCCAA  R: CACGTACAATCCCAACATGC |
| GRANOULE-BOUND STARCH SYNTHASE2 | XM_006491301 | F: GGCATACTTCCCTTATCCCTTGT  R: TTGGCACTTTTGTACATCCCTTT |
| SUGAR SIGNALING | XM_006490335.2 | F: CCATGGCTTCTGATTCTGGC  R: CGCTCCTCTTGACTTGCTTC |
| SUSY6 (sucrose synthase6) | XM_015530185 | F: TCATCACCATTGTTTGGGTCAA  R: CACTTTTGCAACCAATCAAGGA |
| TERPENE SYNTHASE 14 | XM_006467882 | F: CCATCCTTACCATCTTGATTCTCA  R: TTCTTCGGCTCTGGGATGAC |
| TERPENE SYNTHASE 21 | XM_006494650.2 | F: AAGCACGAAAGCCGATGTG  R: TGATCACCCCAAATGCTAGGA |
| TGA5 | XM_006465700 | F: CTGGGAACGTGGCAAACTAT  R: AGAGCACGTAGTCGGGAGAA |
| WRKY33 | XM_006481063 | F: ATCCGGGTCCCAAAATCTAC  R: ATAGAGCCAAAGACGAGCCA |
| WRKY54 | XM_006435880.1 | F: GGAAGATTCCGGGGAGAGTA  R: TTTCTCCATGCATGACCATC |
| WRKY59 | XM_006464429 | F: GATGGCTTCAAATGGAGGAA  R: GAATTAGGCTGCAAGGTCCA |
| ACS-1 (ACC synthase 1) | XM_006477260 | F: GGAACCCAAGCCAATCAGTAGT  R: AACCACCCCTTGTAACGGTACA |
| TERPENE SYNTHASE 3 | XR_371274 | F: GGGAGAATCGTATGCCAGACA  R: GCTGTAGCATCGCGCTTACTT |
| Ent-kaurenoic acid oxidase | XM_006464276 | F: tcgtggatagcttcgtctcc  R: catgaccattgacaggagcg |
| HSP82 (Heat Shock Protein 82) | XM_006490568 | F: TCCTTCGTGAACTCATCAGCAA  R: GTCAGGCTCTCGAATCGAATCT |
| ABC TRANSPORTER | XM_006481762 | F: GCTTCTTTCTGGAAACAGTATTGGT  R: TGGCAGTCATGAGAAATCGAAT |
| SULFOTRANSFERASE 1 | XM_006484190 | F: GGCCCTTTTGGGATCATATT  R: TTTTCCCGTTTCTGTTGACC |
| 12-OXOPHYTODIENOATE REDUCTASE 1-LIKE | XM_006494159 | F: GCTACCACCACCATTCCTCT  R: TACCCTTGAGCAGTGTCGTT |
| GIBBERELLIN-RESPONSIVE PROTEIN | XM_006473594 | F: CAAGGGCAGACCTCAGTGTTG  R: GCGCCCGTTGCAATTG |
